# Quantifying Heterogeneous Impacts of Geolocated mHealth Tracing on Spatiotemporal Transmission of Emerging Infections

**DOI:** 10.1029/2026GH001838

**Published:** 2026-08-10

**Authors:** Zhifeng Cheng, Hao Liang, Fei Gao, Xuyao Chen, Xiao Liu, Yuping Zhou, Xu Chen, Hui Jiang, Samantha Cockings, Andrew Tatem, Shengjie Lai, Chu He

**Affiliations:** ^1^ WorldPop School of Geography and Environmental Science University of Southampton Southampton UK; ^2^ Jiangsu Provincial Center for Disease Control and Prevention Nanjing China; ^3^ Beijing Chest Hospital Capital Medical University, & Beijing Tuberculosis and Thoracic Tumor Research Institute Beijing China; ^4^ School of Geography and Environmental Science University of Southampton Southampton UK; ^5^ Institute for Life Sciences University of Southampton Southampton UK

**Keywords:** mobile health, COVID‐19, China

## Abstract

Digital health tools, including mobile device location‐based health (mHealth) applications, have been adopted to augment conventional contact tracing during recent epidemics and pandemics, yet quantitative evidence about their added epidemiological value remains limited. Using aggregated, privacy‐preserving mHealth data from six Chinese cities (July 2021–May 2022), we modelled transmission dynamics of emerging respiratory infections to quantify the heterogeneous effects of mHealth‐triggered movement restrictions and risk‐based quarantines across viral variants. We found that the mHealth‐assisted interventions, together with population‐wide lockdowns, could rapidly reduce the effective infection rate and drive daily incidence toward zero within two weeks, which was rarely achieved through traditional contact tracing alone. Scenario simulations further showed that in the absence of mHealth interventions, infections consistently propagated to connected cities across a wide range of epidemiological assumptions, contradicting the reality. These results suggest that early integration of geolocated mHealth tools with traditional epidemiological investigations enhances outbreak containment at both local and regional scales, providing quantitative, spatially explicit evidence to inform scalable mHealth integration strategies for future infectious threats with pandemic potential.

## Plain Language Summary

Timely identifying infected individuals and quarantining people who have been in close contact with them is crucial for breaking transmission chain during infectious disease outbreaks. However, traditional epidemiological investigations can be slow and may miss contacts. Using aggregated mobile device location‐based health (mHealth) data, this study quantified the effects of digital tracing tools in containing emerging respiratory infections. We found that the timely implementation of geolocated mHealth tracing substantially strengthens traditional contact tracing approaches, especially for more contagious and severe viruses, helping prevent widespread transmission and reducing the risk of outbreaks escalating beyond control.

## Key Points

- Mobile health (mHealth) tracing complements epidemiological investigation to remove high‐risk individuals from contact networks
- The mHealth effectiveness is strongly time‐dependent, with short delays sharply increasing infections and undermining control measures
- Timely mHealth implementation prevents geographic spillover of infections to interconnected regions along routine mobility pathways

## 1 Introduction

Human movement and physical contact facilitate the spread of acute human‐to‐human transmissible infectious diseases (Badr et al., 2020; Balcan et al., 2009; Charu et al., 2017). Mapping population mobility and contact patterns in communities at high risk is critical for interrupting the spread, predicting disease dynamics, and assessing intervention effectiveness (Kraemer et al., 2020; Lai et al., 2020; Zhou et al., 2020). In various outbreaks over the past decades, such as SARS, MERS, and Ebola, epidemiological investigation focusing on contact tracing and case detection has been widely adopted to retrieve local contact networks and guide targeted interventions (Kang et al., 2016; Pang et al., 2003; Riley et al., 2003; Sun & Viboud, 2020; Svoboda et al., 2004). Subsequent public health measures isolate not only infected individuals but also their close contacts, thus cutting off specific transmission pathways associated with elevated infection risks.

Despite its accuracy and epidemiological value, survey‐based contact tracing can be constrained by limited timeliness and spatial coverage when facing highly transmissible pathogens with pandemic potential (Kwok et al., 2019). First, its time efficiency depends largely on the availability of trained personnel and resources for public health investigations. It may take several days to identify all close contacts of a single case, which often falls behind the pace of transmission. Second, traditional investigations rely on the retrospective recall by infected individuals, which may be incomplete or biased. Missed contacts can remain infectious in the community, triggering secondary waves and undermining outbreak control.

In this digital age, mobile device location‐based health techniques (mHealth) promise to compensate for the shortcomings of traditional investigations by enabling digital contact tracing and being integrated with common geospatial and mobility data. During the COVID‐19 pandemic, digital contact tracing applications were rapidly deployed across countries (Ferretti et al., 2020; Pollmann et al., 2021), including the NHS COVID‐19 app in England and Wales (Ferretti et al., 2024; Wymant et al., 2021), the Corona 100m app in South Korea (Kim, 2021), and TraceTogether in Singapore (Chow et al., 2023). These applications recorded individuals’ health statuses, signalled potential contacts and infections, and enforced travel restrictions on targeted high‐risk groups. Empirical evidence from the UK revealed that the secondary attack rate (6%) among app‐notified close contacts is similar to those identified through manual tracing, thus an increasing adoption of the app could reduce overall transmission (Wymant et al., 2021). However, few studies have quantitatively examined how digital tracing alters transmission dynamics across space and time, particularly under heterogeneous mobility patterns and variant‐specific epidemiological characteristics.

In this study, we used the aggregated mHealth statistics for multiple outbreaks in Jiangsu province, China, to assess the epidemiological impact of geolocated mHealth contact tracing in controlling SARS‐CoV‐2 infections across different viral variants in 2021–2022. To do so, we developed a two‐step epidemiological modelling framework to reconstruct the disease dynamics and quantify the relative contributions of precautionary non‐pharmacological interventions (NPIs), traditional epidemiological investigation, and mHealth‐enabled digital screening. While the framework does not aim to fully isolate the effects of individual interventions, it allows us to compare their respective impacts under diverse outbreak scenarios. By explicitly incorporating variant‐specific epidemiological characteristics, we evaluated the relative importance of mHealth tracing, together with other interventions, to outbreak containment and assessed how delayed or absent implementation alters epidemic trajectories across space and time.

## 2 Methods

### 2.1 Study Areas and Outbreaks

**Figure 1:**
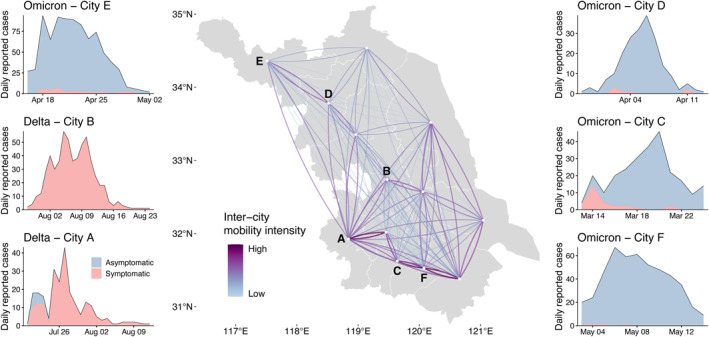
Study locations and epidemiological curves of outbreaks, July 2021–May 2022. The inter‐city mobility network presents the average value of Baidu migration index over the first half of 2022, with line widths and colours proportional to the intensity. The line plots on both sides show the daily number of reported cases in each outbreak, where symptomatic infections are coloured in light red and asymptomatic infections are coloured in light blue.

This study incorporates six SARS‐CoV‐2 outbreaks occurring in different prefectural cities of Jiangsu province, China, between July 2021 and May 2022 (Figure 1). Each outbreak involved at least 200 confirmed infections and lasted for a minimum of 15 days (Table S1 in Supporting Information S1), ensuring sufficient temporal depth for reconstructing transmission dynamics. Two of them were caused by the Delta variant in 2021 (City A and City B) and the remaining four outbreaks were associated with the Omicron variant in 2022 (City C to City F). Variant‐specific epidemiological parameters used in the modelling are summarised in Table 1.

**Table 1:** Variant‐Specific Epidemiological Parameters Used in the Transmission Modelling

Parameters	Values	Reference
Delta		
$R_{0}$	4.90 (95% CI: 3.10, 6.50)	Kang et al. (2022)
Latent period	4.40 (95% CI: 4.24, 4.63)	Li et al. (2024)
Incubation period	5.04 (95% CI: 4.83, 5.33)	
Infectious period	6.00 (IQR: 5.00, 8.00)	Garcia‐Knight et al. (2022)
Secondary Attack Rate	5.5%	Jin et al. (2023)
Omicron		
$R_{0}$	9.50 (IQR: 7.25, 11.88)	Liu & Rocklöv (2022)
Latent period	2.50 (95% CI: 2.27, 2.76)	Li et al. (2024)
Incubation period	3.42 (95% CI: 3.00, 3.89)	
Infectious period	5.16 (95% CI: 4.18, 6.14)	Wu et al. (2023)
Secondary Attack Rate	3.2%	Shang et al. (2023)

CI–Confidence Interval; IQR–Interquartile Range.

### 2.2 mHealth Code System and Classification of Exposure Risk

**Figure 2:**
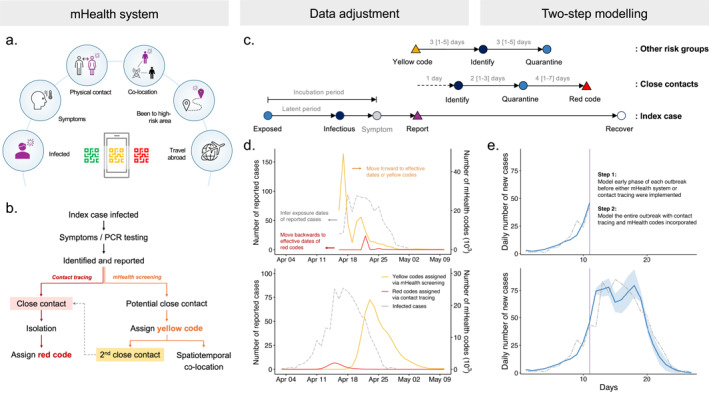
Schematic workflow of the geolocated mHealth code system and modelling framework. (a) Data sources underpinning the mHealth code system. (b) The workflow of mHealth code assignment for individuals at potential exposure risk linked to each index case. (c) The timeline for mHealth code assignment and the corresponding quarantine of risk groups. (d) The strategy for adjusting delays in mHealth code assignment and taking effect, as well as delays in case identification and reporting. (e) The two‐step SEIR‐based modelling framework that first estimates population‐wide transmission dynamics under background NPIs and vaccination, then incorporates mHealth‐enabled risk‐based isolation to estimate its marginal impact on top of these measures. (d) and (e) are illustrated using the Omicron outbreak in City E.

The mHealth codes were developed by the provincial health department to integrate multi‐source information from clinical diagnoses, mobile phone positioning data, customs entry records, and domestic transportation systems including railway, airline, and highway travel (Figure 2a). This system functioned as a population‐scale exposure surveillance and risk stratification tool during the outbreaks. Residents assigned with a green code were permitted unrestricted travel, indicating a low risk of infection. However, the system assigned yellow or red codes to individuals identified as having elevated infection risk based on either epidemiological investigations or automated digital screening. (https://www.jszwfw.gov.cn/col/col169532/index.html).

Under traditional epidemiological investigation, close contacts of confirmed or asymptomatic cases were identified through field surveys conducted by public health personnel and were assigned red mHealth codes. Digital screening complemented manual investigations by incorporating spatiotemporal exposure and behavioural risk indicators. Specifically, individuals were assigned mHealth red or yellow codes according to the following criteria (https://www.nhc.gov.cn/jkj/c100063/202105/b0ee9fbc75954d99a272c0ae6e9a258a/files/1734001309345_45015.pdf):

- Travelled to high‐risk areas within the preceding 14 days (red code);
- Travelled to medium‐risk areas within the preceding 14 days (yellow code);
- Presence of suspected symptoms such as fever and shortness of breath (yellow code);
- Identification as a close contact of a close contact (secondary close contact) (yellow code);
- Spatiotemporally co‐location with confirmed cases based on mobile positioning data (yellow code).

A yellow code denoted a moderate risk of infection and triggered restrictions on access to public spaces, alongside requirements for polymerase chain reaction (PCR) testing. A red code represented confirmed infection or high likelihood of being infected, necessitating hospitalisation or centralised quarantine. Both yellow and red codes were typically valid for 14 days, covering the estimated incubation and infectious periods of the disease.

### 2.3 Timing and Functional Role of mHealth Codes

Figure 2b shows the workflow of mHealth code assignment. Individuals infected by each case were identified through either epidemiological investigation or the mHealth system. Conventional field investigations typically required several days to identify all close contacts of each index case, during which only a subset of contacts could be identified and assigned red codes immediately, i.e., within one day. In contrast, the mHealth system enabled rapid identification of broader at‐risk populations through big‐data screening. Individuals flagged through this process were preemptively assigned with yellow codes at or near the time the index case was reported, even before their specific exposure status was confirmed. In the following days, these individuals were progressively reclassified based on additional information, such as whether they were close contacts missed in previous epidemiological surveys (switched to red codes thereafter), secondary close contacts, or persons spatiotemporally co‐located with confirmed cases. Therefore, yellow codes were theoretically assigned more rapidly than red codes. Red codes were typically assigned after the close contacts had been formally identified and placed under isolation, while the early restriction of movement among broader at‐risk populations could be attributed primarily to the timely assignment of yellow codes, which effectively limited public‐space access and reduced opportunities for onward transmission.

In this study, we empirically inferred the daily number of people under quarantine by moving yellow codes forward in time and red codes backwards in time from their assignment dates to the dates at which the related quarantine measures were actually enforced. Figure 2c illustrates the workflow of time shift for red and yellow codes. Following instructions from the provincial CDC, individuals assigned with yellow codes typically required an average of three days to confirm their risk levels (e.g., close contacts or not), followed by another three days to implement home or centralised quarantine. Individuals with red codes were processed slightly faster, which took two days on average for isolation or hospitalisation after clear identification, and an additional four days on average for red code assignment (a subsequent measure that would not affect the fact that they had already been isolated). To sum up, we moved the assignment date of yellow codes forward by six days on average and the assignment date of red codes backwards by six days on average. These opposite approaches ensure that red codes take effect earlier than yellow codes, reversing the patterns shown in the original data (Figure 2d), thus more closely reflecting the actual situation of first isolating close contacts during an outbreak.

In this paper, all analyses were conducted using the statistical and aggregated mHealth data, which summarised daily code transitions at the city level. No individual‐level records were accessed or analysed. To further mitigate privacy concerns, address potential public sensitivities, and avoid regional stigmatisation, we withheld specific city names and replaced them with proxy identifiers in figures and analyses.

### 2.4 Additional Epidemiological and Mobility Data

Other data for analysis include those related to epidemiological surveillance records, mobility indicators, NPI policies, and vaccination. We gathered the daily number of reported infections, including confirmed cases and asymptomatic infections from official announcements in each prefectural city, with referrals from asymptomatic to confirmed cases accounted for (https://www.jscdc.cn/xxgk_1641/yqdt/). The city‐level population mobility was obtained from the Baidu Migration data portal (https://qianxi.baidu.com/), which summarises people’s intra‐city outdoor activity intensity and inter‐city movement index. The data were collected based on GPS positioning when Baidu users opt in to share their locations in location‐based services. The intra‐city outdoor activity intensity relates to the percentage of intra‐city movement compared to the number of residents, while inter‐city movement index relates to the magnitude of people inbound/outbound cities. The requirements on facemask wearing were collected from the government website of each city and were processed into an ordinal scale representing the level of policy stringency. Vaccination coverage data at the province level were collected from the website of the Chinese National Health Commission (https://www.nhc.gov.cn/cms‐search/downFiles/ed04eda3068846e2bd746b4a2dda229d.pdf).

### 2.5 Inferring Exposure Dates of Reported Cases

Infections typically occur prior to case identification and reporting. To reconstruct the transmission dynamics in models, we extrapolated the daily number of new infections from the officially reported daily new cases, thereby accounting for delays between exposure and reporting (Lai et al., 2020). The total delay was modelled as the sum of two components, and we treated symptomatic and asymptomatic cases as two separate groups. For the first step, we sampled the incubation period for each symptomatic case from a predefined distribution derived from previous studies (Table 1); while for asymptomatic cases, we sampled the latent period. This reflects the well‐documented time window between exposure and symptom onset or being infectious, both of which mean the infected are identifiable from either typical symptoms, massive testing, or contact tracing. Next, we included the reporting delay, defined as the time lag from identification to being reported. This lag was set at three days for cases reported at the onset of an outbreak and then decreased progressively (by one day for every day of epidemic development) as response measures strengthened (Lai et al., 2020). This parameterisation reflects administrative and logistical delays during the early response phase, when diagnostic and reporting systems were not yet fully streamlined. We required the reporting delay to have a minimum value of one day, coinciding with the next‐day reporting mechanism when the surveillance capacity for outbreak containment has stabilised in the later stages of the epidemic. The inferred daily number of new infections was calculated as the median of 1000 times stochastic simulations incorporating random draws of incubation periods and latent periods (Figure S1 in Supporting Information S1).

### 2.6 Two‐Step Compartmental Modelling Framework

Transmission dynamics could be jointly shaped by population‐wide exposure conditions and the efficiency with which high‐risk individuals are identified and isolated. In the study settings, population‐level transmission was primarily influenced by NPIs, including facial masking and reductions in population mobility and contact, as well as baseline immunity from vaccination. In contrast, the removal of individuals at elevated infection risk depended on the timeliness and coverage of traditional epidemiological investigation and geolocated mHealth code‐enabled screening. Leveraging the temporal lag between infection events and the time when mHealth codes started taking effect (i.e., the implementation of targeted quarantine measures), we designed a two‐step compartmental modelling framework to quantify the relative and marginal effects of mHealth codes on top of population‐wide measures under empirically grounded assumptions and data constraints (Figure 2e).

In the first step, we modelled the early phase of each outbreak, typically spanning 11 to 18 days, during which infections occurred prior to the first case reported and before either mHealth codes or large‐scale epidemiological investigations were implemented. This period represents transmission under background public health conditions, shaped by general behavioural responses, baseline NPIs, vaccination coverage, and population mobility, but without targeted removal of exposed individuals. We adopted a classic Susceptible‐Exposed‐Infectious‐Removed (SEIR) framework to describe disease progression during this phase as follows:

(1) $$\beta_{0}=R_{0}\gamma$$

(2) $$\beta_{0,t}^{\prime }=\beta_{0}\cdot \alpha \cdot {\text{Mobility}}_{t}\cdot \left(1-{b_{1}\cdot \text{Mask}}_{t}\right)$$

(3) $$N=S_{t}+E_{t}+I_{t}+R_{t}$$

(4) $$S_{0}=N\cdot \left(1-V_{t0}\right)$$

(5) $$S_{t+1}=S_{t}-\beta_{0,t}^{\prime }{\;I}_{t}S_{t}/N$$

(6) $$E_{t+1}=E_{t}+\beta_{0,t}^{\prime }\;I_{t}S_{t}/N-\delta E_{t}$$

(7) $$I_{t+1}=I_{t}+\delta E_{t}-\gamma I_{t}$$

(8) $$R_{t+1}=R_{t}+\gamma I_{t}$$

where $R_{0}$ is the basic reproduction number. δ is the reciprocal latent period. γ is the recovery rate, i.e., the reciprocal infectious period. $V_{t0}$ is the proportion of the population who were vaccine‐protected at the start of each outbreak. Given the short duration of each outbreak and the fact that the majority of populations have not been infected, vaccination coverage and immunity were assumed constant, and waning immunity was not considered. We also accounted for the efficacy of the commonly used COVID‐19 vaccines in China, i.e., Sinovac‐CoronaVac and Sinopharm, and the proportion of the population receiving full doses and booster shots (see Table S2 in Note S2 in Supporting Information S1).

The infection rate $\beta_{0,t}^{\prime }$ between the susceptible and the infected populations was specified as a time‐varying function to reflect changes in population‐level exposure, jointly impacted by background public health measures. ${\text{Mask}}_{t}$ is the daily proportion of the population wearing a mask, where its efficiency for preventing indoor transmission $b_{1}$ was set as 25%, following previous studies (Coclite et al., 2020; Ge et al., 2023). The relative intensity of intra‐city movement ${\text{Mobility}}_{t}$ with a range of 0‐1 was indicated by the Baidu Mobility Index, which captured relative changes in movement intensity inside cities compared with pre‐outbreak baselines (i.e., the average value during the corresponding weekdays over the three weeks preceding each outbreak). The coefficient α was estimated to represent the proportional variation in transmission attributable to mobility changes, allowing us to measure the effect of spatial movement constraints on transmission risk.

In the second step, the mobility‐ and NPI‐adjusted infection rate estimated in the first step was fixed and applied to the entire outbreak period, during which traditional contact tracing and mHealth code interventions were active. This step explicitly models the identification and quarantine of high‐risk individuals and their removal from the transmission process. Here, we assumed new infections among close contacts could be inferred by the secondary attack rate (SAR), defined as the probability of transmission between an infectious individual and a susceptible close contact.

For each simulation day, we first estimated the daily effective infection rate $\beta_{t}$ by combining the infection rate $\beta_{0,t}^{\prime }$ with the rolling number of individuals assigned mHealth codes over the preceding 14 days, corresponding to the standard duration of code validity. We then reduced the exposed compartment (*E*) by subtracting the number of newly identified close contacts multiplied by the SAR, assuming that these individuals were quarantined and did not contribute to onward transmission from the following days. This captures the cumulative effect of rapid digital screening and slower manual contact tracing in shrinking the pool of individuals capable of generating secondary infections. The compartmental model can be specified as follows:

(9) $$\beta_{t}=\beta_{0,t}^{\prime }\cdot e^{-\lambda_{\text{Code}}\cdot {\text{Code}}_{t}/I_{t}}$$

(10) $$N=S_{t}+E_{t}+I_{t}+R_{t}+Q_{t}$$

(11) $$S_{0}=N\cdot \left(1-V_{t0}\right)$$

(12) $$S_{t+1}=S_{t}-\beta_{t}{\;I}_{t}S_{t}/N$$

(13) $$E_{t+1}=E_{t}+\beta_{t}\;I_{t}S_{t}/N-\delta E_{t}-\text{SAR}\cdot {\text{Close}}_{t}$$

(14) $$Q_{t+1}=Q_{t}+\text{SAR}\cdot {\text{Close}}_{t}$$

(15) $$I_{t+1}=I_{t}+\delta E_{t}-\gamma I_{t}$$

(16) $$R_{t+1}=R_{t}+\gamma I_{t}$$

where $\beta_{t}$ is the effective infection rate on day *t*, ${\text{Close}}_{t}$ represents the daily number of newly identified close contacts, $Q_{t}$ is the quarantine compartment and individuals entering it are assumed not to contribute to onward transmission. ${\text{Code}}_{t}$ denotes the rolling number of individuals assigned mHealth codes from day *t*‐13 to day *t*, and $\lambda_{\text{Code}}$ quantifies the marginal effect of mHealth‐enabled digital screening on reducing transmission. The ratio ${\text{Code}}_{t}/I_{t}$ reflects the relative coverage of mHealth restrictions per infectious individual. We further conducted a sensitivity analysis using ${\text{Code}}_{t}$ directly, without normalisation by $I_{t}$. While the estimated parameter values differed between the two approaches, overall model performance remained comparable (Figure S2 and Table S3 in Supporting Information S1).

We acknowledge that, although our two‐step modelling framework was designed to measure the marginal effect of mHealth codes on top of other control measures, mHealth interventions might also directly influence human mobility (e.g., by restricting movement among coded individuals), which could in principle lead to underestimation of their impact. To assess the robustness of this design to this potential overlap, we first tested an extreme scenario in which the intra‐city mobility index was recalibrated, assuming that all individuals identified by the mHealth system were fully isolated. This yielded very similar goodness‐of‐fit and parameter estimates compared with the main analysis (Figure S3 and Table S4 in Supporting Information S1). We then refitted the model using within‐outbreak interrupted time series specifications with lagged mobility data. These lagged models generally showed worse fit or produced substantial changes in the estimated mHealth effect (Figures S4, S5 and Table S5 in Supporting Information S1). Taken together, these analyses suggest that while some overlap is unavoidable, the potential conflation between mobility restrictions and mHealth effects is limited, and the framework captures their complementary contributions to transmission reduction.

### 2.7 Model Fitting

The two‐step model was fitted using a stochastic Markov process with maximum likelihood estimation. For the first modelling step, the primary unknown parameter was the coefficient α, representing how residual movement intensity scaled the infection rate. This parameter was constrained to the interval [0, 1]. Initial values were sampled from a uniform distribution over this range, and the model was fitted iteratively using 1000 randomly sampled starting points. Model performance was evaluated by comparing the reported and estimated daily new infections using the root mean squared error (RMSE) as the likelihood metric. After each iteration, we retained the 100 parameter values with the smallest RMSE, and then constructed a normal distribution from their mean and standard deviation. This distribution served as the probability distribution of α for the next iteration. This process was repeated until convergence, defined as stabilisation of RMSE across successive iterations.

In the second step of model fitting, the coefficient α estimated from step one was fixed, and the full outbreak model incorporating traditional contact tracing and mHealth code interventions was fitted. The key unknown parameter $\lambda_{\text{Code}}$ quantified the marginal reduction in transmission attributable to mHealth‐enabled digital screening. Initial values for $\lambda_{\text{Code}}$ were sampled from a wide range of 10^−5^ to 10^2^ with equal probability, to ensure adequate exploration of plausible effect sizes. The same iterative fitting and convergence criteria were applied as the first step.

We ran stochastic models 1,000 times for both model fitting and scenario simulations. Variant‐specific epidemiological parameters, including $R_{0}$, the latent period 1/δ, the infectious period 1/γ, and SAR, were obtained from the published literature and are summarised in Table 1. We took the viral shedding time to indicate the infectious period. To reflect biological variability across variants, in each run, latent and infectious periods were resampled from their respective distributions, while $R_{0}$ and SAR were held constant. Model outputs are presented as medians with interquartile ranges. Sensitivity analyses were conducted by varying $R_{0}$ and latent periods across plausible ranges to assess robustness to uncertainty in baseline transmissibility (See Results).

### 2.8 Scenario Simulations

To reveal the additive role of geolocated mHealth codes in containing potential large‐scale outbreaks, we conducted counterfactual scenario simulations in which mHealth code interventions were absent or implemented with systematic delays. These scenarios were designed to characterise the incremental effect of timely, spatially informed risk identification while retaining other public health measures observed during the actual outbreaks.

In the no‐mHealth scenario, we removed the mHealth‐related terms from the second modelling step and applied the mobility‐adjusted infection rate estimated in the first step throughout the outbreak period. This scenario represents control efforts relying on population‐wide NPIs and traditional contact tracing alone. In delayed‐implementation scenarios, we retained the full model structure, but the daily number of individuals assigned mHealth codes was postponed by one to seven days. These delays reflect possible lags between initial case detection and full deployment of digital screening systems. To avoid overestimating mHealth effects, we also tested the scenario where traditional epidemiological investigation was assumed to continue with human resource constraints considered (see Note S5 in Supporting Information S1).

In addition, to simulate the geographical spillover of the virus across cities, we extended the model to include inter‐city population movement after completing the infection‐related processes for each day. Individuals in the exposed compartment ($E_{t}$) were allowed to move between cities. For each exposed individual, Bernoulli trials were conducted for all possible destination cities (including remaining in the same city), with movement probabilities parameterised by inter‐city mobility patterns. Specifically, the volume of travel was inferred by combining the Baidu Inter‐city Mobility Index and the estimated scaling factor between the index and actual population movement(Yuan et al., 2020); the probability of travel was set equal to the proportion of Baidu users travelling between the two cities(Lai et al., 2020). This approach allowed spillover risk to emerge endogenously from the interaction between infection burden and routine mobility networks, rather than being imposed exogenously.

Each scenario was simulated 1000 times by taking different sampled parameters. Simulation outputs were summarised using median trajectories and interquartile ranges. Comparisons across scenarios allowed assessment of how intervention timing interacts with variant transmissibility and population mobility to influence outbreak trajectories. All data collation, modelling, and analyses were conducted using R (version 4.3.2; R Foundation for Statistical Computing).

## 3 Results

### 3.1 Model Performance and Goodness‐of‐Fit

**Figure 3:**
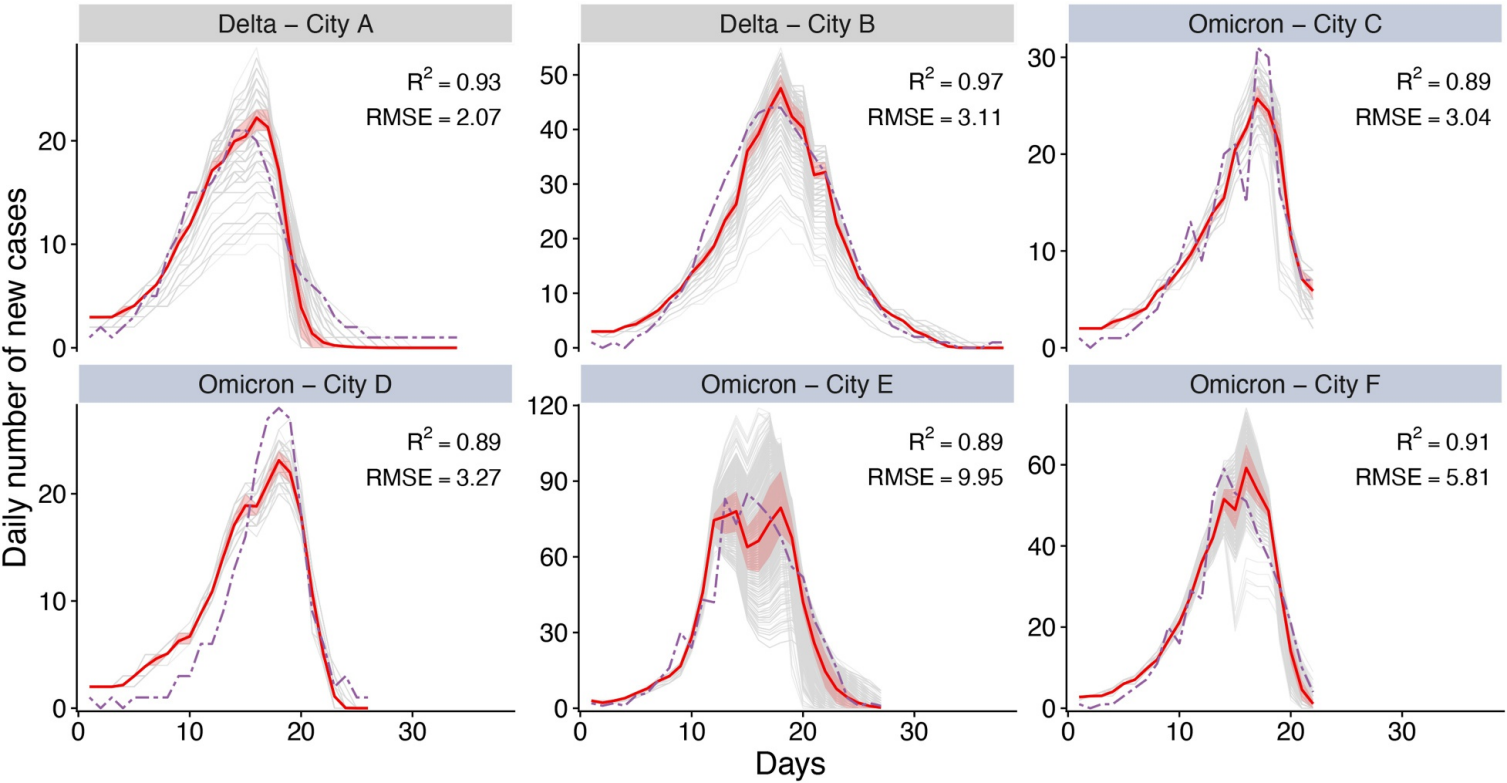
Comparison of daily new infections between reported data and model estimates. The purple dashed lines represent the number of reported cases. For each city, the model was fitted 1000 times with stochastic sampling of latent and infectious periods, shown by lines in light grey. The red solid lines denote the median model estimates, and the shaded light red areas indicate the 25th–75th percentile range.

We applied the two‐step modelling framework to each of the six outbreaks, sampling the latent period and infectious period 1000 times per outbreak and using the median estimates to assess the goodness‐of‐fit. Across all cities and variants, the estimated daily new infections closely match the reported incidence curves (Figure 3). The coefficient of determination (*R* ^2^) ranges from 0.89 to 0.97, and the RMSE ranges from 2.07 to 9.95, depending on outbreak size and duration. Slightly larger RMSE values are observed in outbreaks with higher cumulative infection counts, reflecting greater stochastic variability during periods of intense transmission. The close agreement between estimated and observed incidence supports the validity of the model for assessing intervention effects across different spatial and temporal contexts.

### 3.2 Differential Contributions of NPIs and mHealth Codes

**Figure 4:**
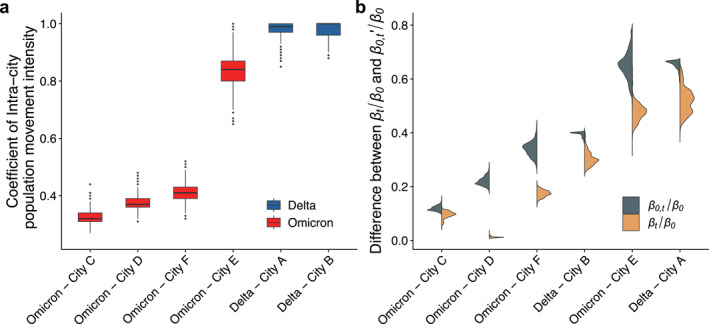
Estimated relative contributions of intra‐city mobility reductions and mHealth digital screening to transmission control. (a) Distribution of estimated coefficients α from the first modelling step, quantifying how intra‐city mobility intensity scales transmission risk in each outbreak. (b) Relative decline in the daily infection rate one week after three consecutive days of mHealth implementation. The violin plots in dark colours summarise the daily infection rate without mHealth effects, $\beta_{0,t}^{\prime }$, relative to the baseline rate $\beta_{0}$, while light coloured violin plots represent the ratio of the daily effective infection rate with mHealth intervention, $\beta_{t}$, to $\beta_{0}$. For each city, 1,000 model estimations were conducted with stochastic sampling of latent and infectious periods. These estimates reflect the marginal reduction in transmission attributable to mHealth codes on top of concurrent NPIs and vaccination, rather than isolated effects of a single intervention.

The estimated relative contributions of population‐wide NPIs and mHealth digital screening on disease containment vary substantially across viral variants and cities (Figure 4). In the first modelling step, the estimated coefficient α, representing the proportional variation in transmission attributable to intra‐city mobility changes, approaches unity for both Delta outbreaks (median 0.98, interquartile range [IQR]: 0.97, 1.00) (Figure 4a). In contrast, they are markedly lower for Omicron outbreaks, falling below 0.4 in most cities, with the exception of city E (0.84, IQR: 0.80, 0.87). These differences suggest that changes in mobility explain a larger fraction of transmission variability for Omicron, highlighting the pronounced effectiveness of mobility‐based interventions alone in suppressing the spread of more transmissible variants.

Beyond mobility effects, mHealth code implementation results in substantial additional reductions in infection rates across all cities (Figure 4b). Even in cities where mobility restrictions have already suppressed transmission to relatively low levels, the inclusion of mHealth‐based risk identification and quarantine consistently produces further declines in the daily effective infection rate $\beta_{t}$. Overall, $\beta_{t}$ declines markedly a week after three days of mHealth implementation. The largest proportion of reductions in infection rates attributable to mHealth codes ($\beta_{t}/\beta_{0}-{\beta_{0,t}}^{\prime }/\beta_{0}$) are observed in City A during the Delta outbreak (median reduction −13.1%, IQR: −17.1%, −10.6%) and in City D for Omicron (−20.6%, IQR: −21.8%, −19.7%). Note that these estimates should be interpreted as the marginal impact of mHealth codes on top of concurrent NPIs, rather than as fully isolated causal effects of a single intervention.

Taken together, the results indicate that mHealth codes provided a critical layer of targeted intervention that complemented, rather than substituted for, broader population‐wide measures. Their combined effect could rapidly reduce the effective infection rate and drive daily incidence toward zero within two weeks (Figure 3).

### 3.3 Scenario Simulations for Absent and Delayed mHealth Implementation

**Figure 5:**
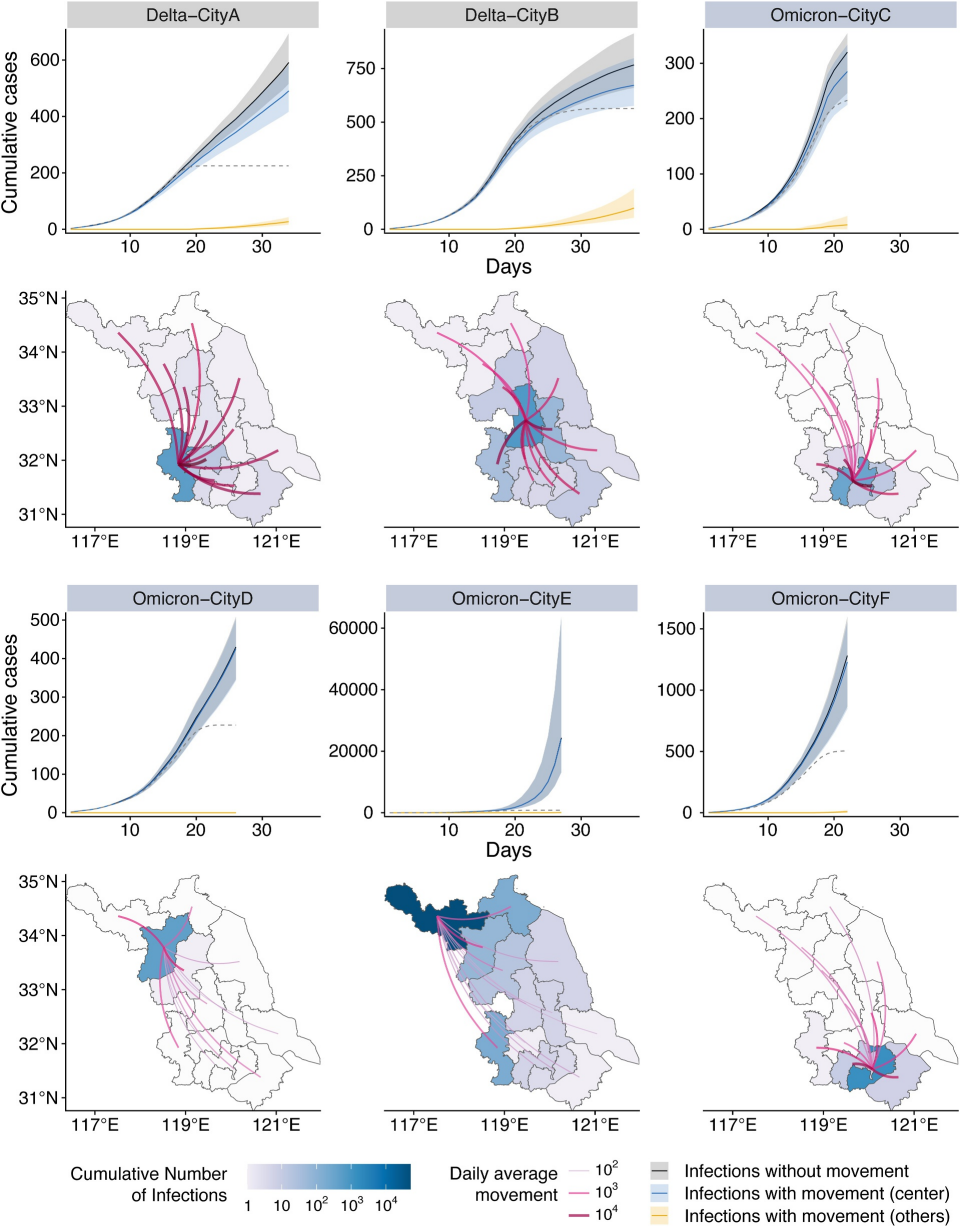
Counterfactual scenario simulations of geographic spillover in the absence of mHealth codes. Simulations were conducted by removing mHealth‐related terms from the SEIR model, while retaining observed NPIs, vaccination, and intra‐city mobility patterns and also incorporating the inter‐city movements. The transmission trajectories for each source city were simulated 1,000 times with stochastic sampling of latent and infectious periods. Black solid lines in line charts represent median simulated incidence without considering inter‐city population movement; solid lines in blue (centre) and yellow (others) represent median simulated incidence in the outbreak city and surrounding cities with inter‐city population movement considered. The shaded areas with corresponding colours indicate the 25th–75th percentile range. Dashed lines represent observed case counts. The corresponding maps show the average simulated incidence with inter‐city population movement considered by the end of each outbreak. The curves divergent from the central city show the average outflow population to other cities during the modelling period, with colours and widths proportional to movement volume.

We next quantified the role of mHealth codes in timely outbreak containment by simulating counterfactual scenarios in which digital screening is absent or delayed. We found that infections would increase continuously throughout the simulation period across all cities in the absence of mHealth codes, with no outbreak achieving elimination of transmission (Figure 5, Figure S6 in Supporting Information S1). Cumulative infections at the end of the simulated outbreaks are substantially higher than observed values, reaching 2.63 times (IQR: 2.29, 3.09) the actual count for the Delta outbreak in City A and 29.53 times (IQR: 16.00, 77.32) for the most severe Omicron outbreak in City E. In addition, postponing mHealth deployment by one to seven days resulted in marked increases in cumulative infections, with longer delays producing nonlinear escalation in epidemic sizes (Figure S9 in Supporting Information S1). Delays of five days or more shifted the epidemic trajectory from a controllable outbreak to sustained community transmission in some cities.

These counterfactual projections represent conservative upper bounds on the marginal effect of mHealth codes as we did not reconstruct the model, nor consider potential changes in other interventions and individual behaviours. In particular, manual contact tracing was not assumed to be continued or enhanced during the period of delayed or absent mHealth deployment. The sensitivity analyses for simulated continued traditional contact tracing could be found in Figure S10 in Supporting Information S1.

### 3.4 Geographic Spillover and Robustness of mHealth Effects Under Virological Uncertainty

Beyond local epidemic control, mHealth code implementation also played a critical role in preventing the spatial spillover of SARS‐CoV‐2 infections to surrounding cities. Under a no‐mHealth scenario allowing inter‐city movement of exposed populations, simulations show that the magnitude and timing of spillover depend jointly on the infection burden in the source city and the volume of population flow to destination cities in the same province (Figure 5). For instance, City A is projected to experience more than ten spillover infections originating from both City B (due to geographical proximity and frequent population exchange) and City E (driven by its larger infection burden and considerable inter‐city mobility), despite the latter's transmission period being 10 days shorter. In contrast, few spillover infections could be found under the scenario in which mHealth system was implemented across all cities (Figures S7 and S8 in Supporting Information S1). These results illustrate that timely mHealth implementation effectively suppressed infections at the source, thereby reducing exportation risk and limiting subsequent regional spread.

**Figure 6:**
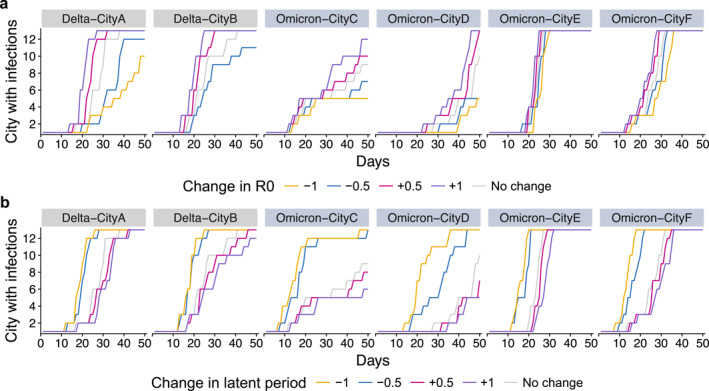
Sensitivity analysis for geographic infection spillover under virological uncertainty. (a) The cumulative number of cities with local infections as the basic reproduction number $R_{0}$ changes. (b) The cumulative number of cities with local infections as the latent period changes. The transmission was simulated by first excluding big‐data‐related mHealth code, then incrementally increasing or decreasing the $R_{0}$ or latent period by a 0.5 step. For each combination of parameter values and source city, 1000 stochastic simulations were performed with resampling of latent and infectious periods, and the lines in the plots show median estimates of each scenario.

We further assessed the robustness of these findings by varying key virological parameters, including $R_{0}$ and the latent period, under no‐mHealth scenarios (Figure 6). Across most parameter combinations, the number of cities experiencing spillover increases over time, confirming that geographic spread would occur under a wide range of epidemiological assumptions in the absence of digital screening. Higher $R_{0}$ values accelerate both local transmission and regional spillover, with infections reaching all 13 prefectural cities in the province substantially earlier when $R_{0}$ increases by 0.5 or more (Figure 6a). Variations in the latent period show a similar pattern: shorter latent periods (−0.5 to −1 days) accelerate spillover timing, while longer latent periods delay spread (Figure 6b). Notably, Omicron outbreaks, particularly those centred in Cities E and F, exhibit faster and more extensive spillover dynamics. Spillover infections eventually reach all cities in the absence of mHealth even under the most conservative assumptions of lower transmissibility or longer latent periods, underscoring the critical role of geolocated mHealth tracing in preventing regional spread regardless of parameter uncertainty.

## 4 Discussion

This study proposes a two‐step compartmental modelling framework to quantify the epidemiological impact of big data screening‐enabled mHealth codes on the containment of emerging respiratory infectious diseases. With the confounding effects of population‐wide NPIs and traditional contact tracing explicitly considered, we show that geolocated mHealth digital screening and associated quarantine can substantially reduce SARS‐CoV‐2 transmission by rapidly lowering the daily infection rate. Importantly, this reduction occurs over a relatively short and epidemiologically meaningful timescale. Early mHealth implementation can interrupt transmission, while without mHealth codes, outbreaks would become difficult to control within the observed study period and could have potentially spread to surrounding cities.

These findings emphasise the importance of physical contact networks and human mobility structures in shaping infectious disease spread. Research has shown that human mobility networks are assortative and hierarchical, with dense local interactions embedded within broader regional and inter‐city connections (Lu et al., 2026). Infectious disease models that explicitly incorporate such real‐world network structures ensure more reliable predictions of epidemic dynamics (Karaivanov, 2020; Zhang et al., 2024). While comprehensive, population‐wide mobility data can offer valuable insights, they are often costly to acquire and slow to operationalise during rapidly unfolding outbreaks (Kostandova et al., 2025). Instead, digitally mediated identification of local contact patterns, particularly index cases and their immediate spatial‐temporal exposure environments, is a more practical and scalable approach for early outbreak containment when facing emerging severe infections (Cauchemez et al., 2011).

From an operational perspective, geolocation‐based mHealth tracing complements traditional epidemiological investigations for two aspects: timeliness and coverage. While manual investigations can precisely identify confirmed close contacts, they are constrained by human resource capacity and retrospective recall. These limitations are exacerbated in dense urban environments and during periods of intense population mobility (Cleary et al., 2025), where many exposure events may occur outside individuals’ social awareness. By contrast, mHealth systems leverage objective spatiotemporal data derived from mobile positioning, transportation records, and health surveillance to quickly flag individuals with potentially elevated exposure risk (Kendall et al., 2023). Informing these groups, temporarily restricting their access to public space, and monitoring their health status could effectively interrupt transmission chains before secondary infections propagate widely, thereby preventing spillover from localised outbreaks to the broader susceptible populations.

We acknowledge some limitations in this study. First, we only incorporated the city‐level mobility indicators and the province‐level vaccination data, which might not fully capture individual‐level contact intensity or heterogeneity in immunity. Although positively correlated, human mobility might not be a perfect proxy for actual interpersonal contact, especially at finer spatial and social scales (Huang et al., 2021). Meanwhile, our model did not account for the potential waning of immunity over time, though this omission is unlikely to substantially affect results given the relatively short duration of the outbreaks studied. Second, although our modelling framework was designed to estimate relative contributions of mobility reductions and mHealth interventions, these measures might still interact in practice. As a result, our estimates should be interpreted as relative, marginal effects of mHealth codes on top of concurrent population‐wide measures, rather than as fully isolated causal effects. The sensitivity analyses summarised in the Methods in Supporting Information S1 were designed to probe this potential overlap and support a cautious, layered interpretation of our findings. Third, our analysis relied on the reported case data, which might be subject to underreporting, particularly when asymptomatic infections predominated. Although the likelihood of systematic underreporting was relatively low under stringent intervention and enhanced surveillance conditions, residual bias could not be entirely excluded. Finally, as an emerging digital health intervention using spatial and temporal information, mHealth systems might exhibit uneven population coverage due to differences in smartphone access, digital literacy, and regional infrastructure. Although mechanisms existed to manage codes for individuals unable to operate smartphones independently, e.g., adults using their phone to present mHealth codes of their children and parents when travelling, disparities associated with the digital divide warrant careful consideration in future research and implementations.

Despite the empirically evidenced contribution, the effectiveness of mHealth codes also depends on institutional coordination, technological precision, and population compliance. During the earlier Delta outbreaks in 2021, mHealth strategies prioritised rapid flagging of all individuals with potential exposure risk, through widespread assignment of yellow codes for secondary close contacts and co‐located persons with index cases, followed by subsequent manual verification and reclassification. While this approach maximised sensitivity, it placed substantial demands on community‐level workers and relied on the spatial resolution of telecommunications infrastructure, which could be limited in dense urban settings (Lu et al., 2026). In subsequent outbreaks, the proportion of yellow codes transitioning to red codes varied and did not necessarily align perfectly with actual control conditions. Consequently, there were discrepancies in the number of identified close contacts within the system, resulting in data gaps, particularly with red code data.

These observations underscore that, at the current stage, big data‐driven digital contact tracing cannot function independently of traditional epidemiological investigations. Instead, effective outbreak control requires a hybrid system in which digital tools enhance, rather than replace, manual tracing, supported by strong inter‐departmental coordination and adequate public health workforce capacity. The performance of such systems is shaped by infrastructure quality, algorithmic precision, governance arrangements, and community engagement, all of which have spatial and social dimensions central to geo‐health.

Nevertheless, this study provides empirical, system‐level evidence quantifying the epidemiological value of geolocated mHealth screening in tracking exposure risks and containing emerging infectious disease outbreaks. By explicitly linking digital tracing to transmission dynamics, mobility patterns, and geographic spillover risk, our findings offer actionable insights for public health agencies seeking to rationally integrate digital technologies with conventional epidemiological practice. Strengthening the inclusivity, precision, and governance of such systems will be essential for preparing for future infectious disease threats with pandemic potential.

## Conflict of Interest

The authors declare no conflicts of interest relevant to this study.

## Supporting information

Supporting Information S1

## Data Availability

The authors declare that all data essential for modelling including daily reported cases, mHealth records, intra‐city mobility index, facemask wearing indicator, and vaccination rate for each outbreak, as well as the codes for model construction and scenario simulation are available at Figshare (Cheng, 2025). The mHealth records were normalized showing relative changes to comply with the data protection requirements of the local Centre for Disease Control and Prevention, and this will not affect the methods and findings of this study.
