# Supplementary material for: Quantifying Heterogeneous Impacts of Geolocated mHealth Tracing on Spatiotemporal Transmission of Emerging Infections
Source: Geohealth. 2026 Aug 10;10(8):e2026GH001838. doi: 10.1029/2026GH001838 (PMC13458281; doi:10.1029/2026GH001838)
Supplement: Supplementary file 1 [file GH2-10-e2026GH001838-s001.pdf]

GeoHealth

Supporting Information for

Quantifying heterogeneous impacts of geolocated mHealth tracing on spatiotemporal transmission of emerging infections

Zhifeng Cheng[1*], Hao Liang[2], Fei Gao[2], Xuyao Chen[2], Xiao Liu[2], Yuping Zhou[2], Xu Chen[2], Hui Jiang[3], Samantha Cockings[4], Andrew Tatem[1], Shengjie Lai[1,5,§], Chu He[2,*,#,§]

Affiliations

[1] WorldPop, School of Geography and Environmental Science, University of Southampton, UK

[2] Jiangsu Centre for Disease Control and Prevention, Nanjing, China

[3] Beijing Chest Hospital, Capital Medical University, & Beijing Tuberculosis and Thoracic Tumor Research Institute, Beijing, China.

[4] School of Geography and Environmental Science, University of Southampton, UK

[5] Institute for Life Sciences, University of Southampton, Southampton, UK.

[*] These authors contributed equally to this work.

[#] Corresponding author.

[§] Senior authors.

Contents of this file

Notes S1 to S5
Figures S1 to S10
Tables S1 to S5

Supplementary Materials

Supplementary Note 1. Back-projection of infection dates from reported case data

The start time, end time, and daily reported cases corresponding to Fig. 1 are listed in Supplementary Table S1.

Table S1. Summary of SARS-CoV-2 outbreaks across six cities included in the modelling.

Variant	City	Date of first reported case	Date of last reported case	Resident population (million)	No. of symptomatic infections	No. of asymptomatic infections
Delta	A	2021-07-20	2021-08-12	9.49	212	22
	B	2021-07-28	2021-08-24	4.58	566	2
Omicron	C	2022-03-13	2022-03-24	5.37	27	196
	D	2022-03-28	2022-04-13	4.98	7	194
	E	2022-04-16	2022-05-12	9.02	36	801
	F	2022-05-03	2022-05-14	7.49	4	467

To infer the timing of infection events, we back-projected infection dates from daily reported cases using stochastic simulations (see Methods). This approach accounts for delays between infection, symptom onset, and case reporting.

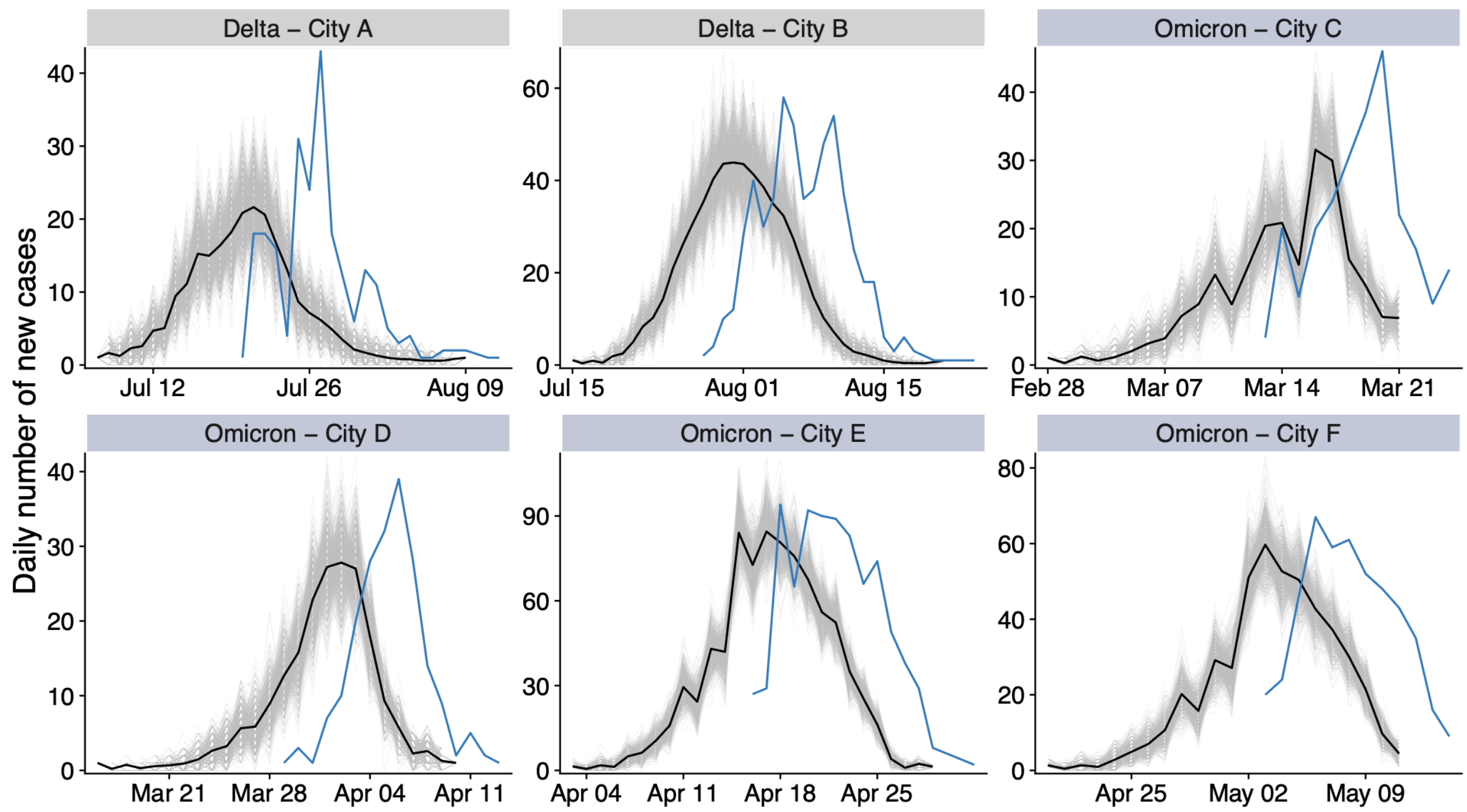

Figure S1. Back-projection of infection dates from reported case data. The blue lines show the daily reported cases. Grey lines are 1000 stochastic simulations of inferred daily new infections. Black lines denote the median estimates across all simulations for each city.

Supplementary Note 2. Vaccine efficacy

Within the SEIR modelling framework, vaccine-induced protection was incorporated by reducing the effective susceptible population based on vaccination coverage at outbreak onset. V_{t0} is the proportion of the population who were vaccine-protected at the start of each outbreak. Given the short duration of each outbreak, vaccination coverage and vaccine-induced immunity were assumed to remain constant over the simulation period, and waning immunity was not considered.

We accounted for the effectiveness of the COVID-19 vaccines predominantly used in China, i.e., Sinovac-CoronaVac and Sinopharm, as well as the proportions of the population receiving full doses and booster shots(Ge et al., 2023). The vaccine efficacy was defined with respect to protection against symptomatic COVID-19, based on estimates from clinical trials (Supplementary Table S2). The effective vaccine protection V_{t0} at outbreak onset was calculated as:

$$V_{t0} = FV_{t0} \overline{\sum_{\iota=1}^{2} e_{f,\iota}} + BV_{t0} \overline{\sum_{\iota=1}^{2} e_{b,\iota}}$$

where $e_{f,i}$ and $e_{b,i}$ denote the efficacy against symptomatic infection of full vaccination and booster doses, respectively, for vaccine i (Sinovac-CoronaVac or Sinopharm). FV_{t0} and BV_{t0} represent the provincial-level proportions of the population that had completed full vaccination and received booster doses, respectively, at the start of each outbreak. The overbar indicates the average efficacy across the two vaccine types, weighted equally due to the lack of city-level vaccine-specific coverage data. Given the short period of simulations for each outbreak, and the fact that the majority of populations have not been infected, the decline in immunity during the outbreaks was not considered.

Table S2. Vaccine efficacy against symptomatic COVID-19 after full vaccination (e_f) and booster shot uptake (e_b). The mean estimates and 95% confidence interval (CI) are shown.

Vaccine	e_f	e_b	Reference
Sinovac-CoronaVac	51%<br>(95% CI: 36 - 62%)	79%<br>(95% CI: 77 - 81%)	World Health Organization. Interim recommendations for use of the inactivated COVID-19 vaccine, CoronaVac, developed by Sinovac: interim guidance, first issued 24 May 2021, updated 21 October 2021, updated 15 March 2022. https://apps.who.int/iris/handle/10665/352472 (2022).
Sinopharm	79%<br>(95% CI: 66 - 87%)	86%<br>(95% CI: 80 - 91%)	World Health Organization. Interim recommendations for use of the inactivated COVID- 19 vaccine BIBP developed by China National Biotec Group (CNBG), Sinopharm: interimguidance, first issued 7 May 2021, updated 28 October 2021, updated 15 March 2022. https://apps.who.int/iris/handle/10665/352470 (2022).

Supplementary Note 3. Robustness check of the two-step modelling framework

3.1 Effect of normalising mHealth code coverage by the number of infectious individuals

In the two-step modelling framework, Equation (9) introduces the component $e^{-\lambda_{Code} \cdot Code_t / I_t}$ to reflect the impact of mHealth-enabled digital screening on daily infection rates. We normalised the number of individuals assigned mHealth code ($Code_t$) by the number of infectious individuals I_t, to account for the potential differences in population size and policy stringency across cities. This normalisation converts the absolute number of mHealth codes into an average level of digital screening coverage per infectious person, improving the comparability of the estimated parameter λ_{Code} across outbreaks and settings.

To assess the robustness of this modelling choice, we re-estimated the model using an alternative specification in which $Code_t$ entered Equation (9) without normalisation by I_t. Model fit and parameter estimates were then compared between the normalised and unnormalised formulations. No substantial differences were observed in the goodness-of-fit metrics or overall model performance (Supplementary Fig. S2 and Table S3)

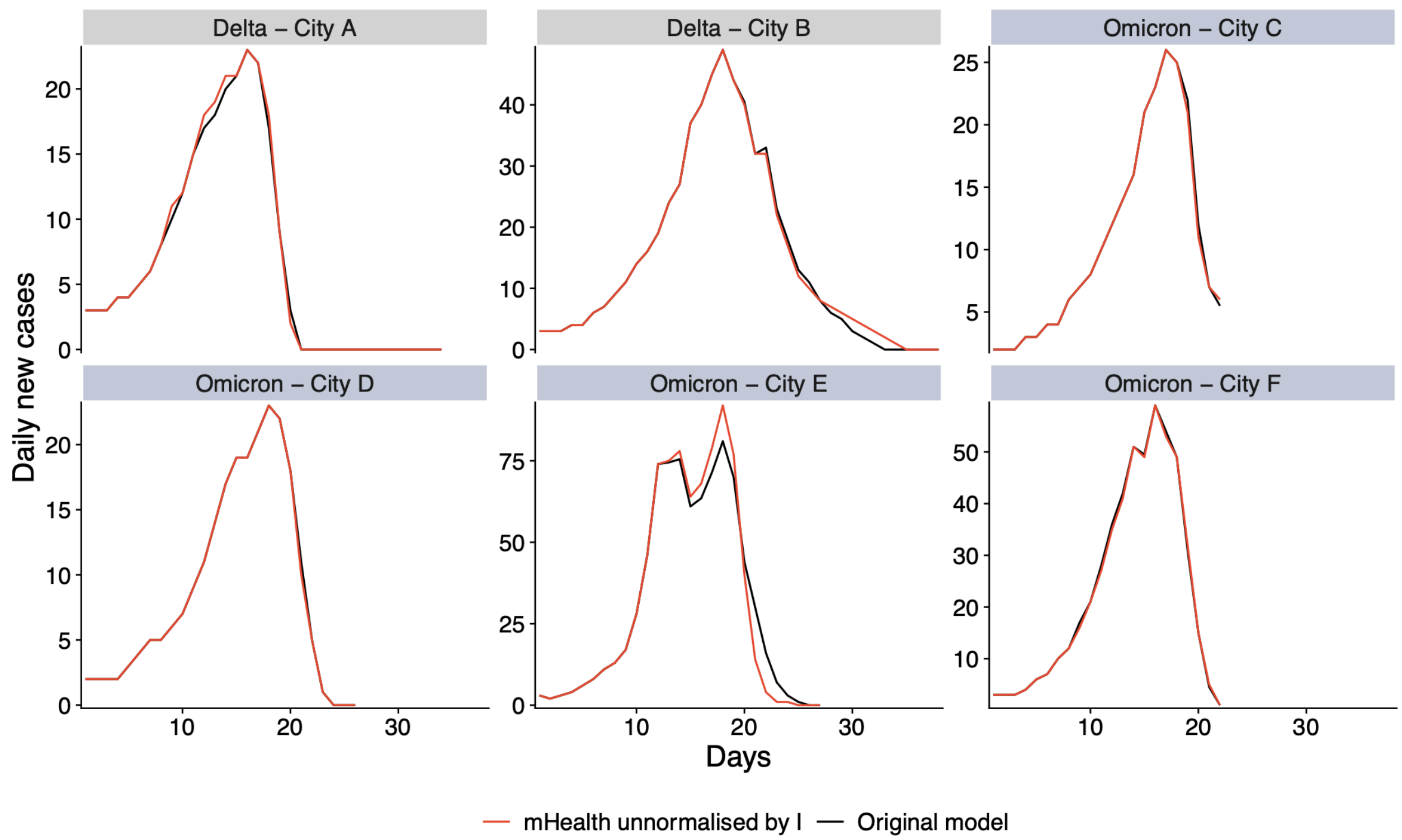

Figure S2. Comparison of model performance between the original specification and an alternative specification in which mHealth code coverage was not normalised by the number of infectious individuals

Table S3. Comparison of estimated parameters between models with and without normalisation of mHealth code coverage by the number of infectious individuals.

**City**	α **(for mobility)**	λ_{Code}	λ_{Code} ($Code_t$ **unnormalized by I)**
Delta - City A	0.98 [0.92, 1.00]	0.0005 [0.0000, 0.0010]	0.000011 [0.000001, 0.000030]
Delta - City B	0.98 [0.92, 1.00]	0.0009 [0.0000, 0.0014]	0.000014 [0.000002, 0.000020]
Omicron - City C	0.33 [0.28, 0.37]	0.0009 [0.0000, 0.0025]	0.000015 [0.000001, 0.000040]
Omicron - City D	0.38 [0.33, 0.44]	0.0170 [0.0090, 0.0235]	0.000248 [0.000167, 0.000304]
Omicron - City E	0.83 [0.72, 0.96]	0.0063 [0.0012, 0.0208]	0.000058 [0.000022, 0.000140]
Omicron - City F	0.41 [0.36, 0.48]	0.0034 [0.0013, 0.0066]	0.000030 [0.000020, 0.000051]

3.2 Influence of mHealth interventions on general human mobility

While we proposed a two-step modelling framework to approximate the contributions of population-wide mobility restrictions and mHealth-enabled digital screening, these interventions might have partially interacted in practice. In particular, individuals assigned mHealth codes might reduce their mobility and contact intensity, potentially influencing estimates of the effect of general population mobility. We therefore conducted sensitivity analyses to assess the robustness of our results to such potential interference.

We first examined whether mHealth interventions could have biased the estimation of the effect of human mobility restriction. As the precise extent to which mHealth code assignment reduced movement and contact intensity among affected individuals was unknown, we considered an extreme (optimistic) scenario in which individuals assigned mHealth codes were assumed to be completely isolated, with no physical contact with household members or the broader community.

Under this assumption, intra-city mobility data for each city were adjusted to better reflect the movement of the remaining populations that were not subject to mHealth restrictions and were free to move. Specifically, the observed mobility index was rescaled by a factor $N/(N - \sum Code_t)$. The modified infection rate was specified as:

$$\beta'_{0,t} = \beta_0 \cdot \left(\alpha \cdot Mobility_t \times \frac{N}{N - \sum Code_t}\right) \cdot (1 - b_1 \cdot Mask_t)$$

We then compared this modified model with the original specification (Supplementary Fig. S3 and Table S4). No substantial differences were observed in model goodness-of-fit or in the estimated parameters, suggesting that the estimated effects of general human mobility on transmission were robust to potential interference from mHealth-based restrictions. These

findings supported the validity of the two-step modelling framework in distinguishing relative contributions of population-wide mobility effects from targeted digital interventions.

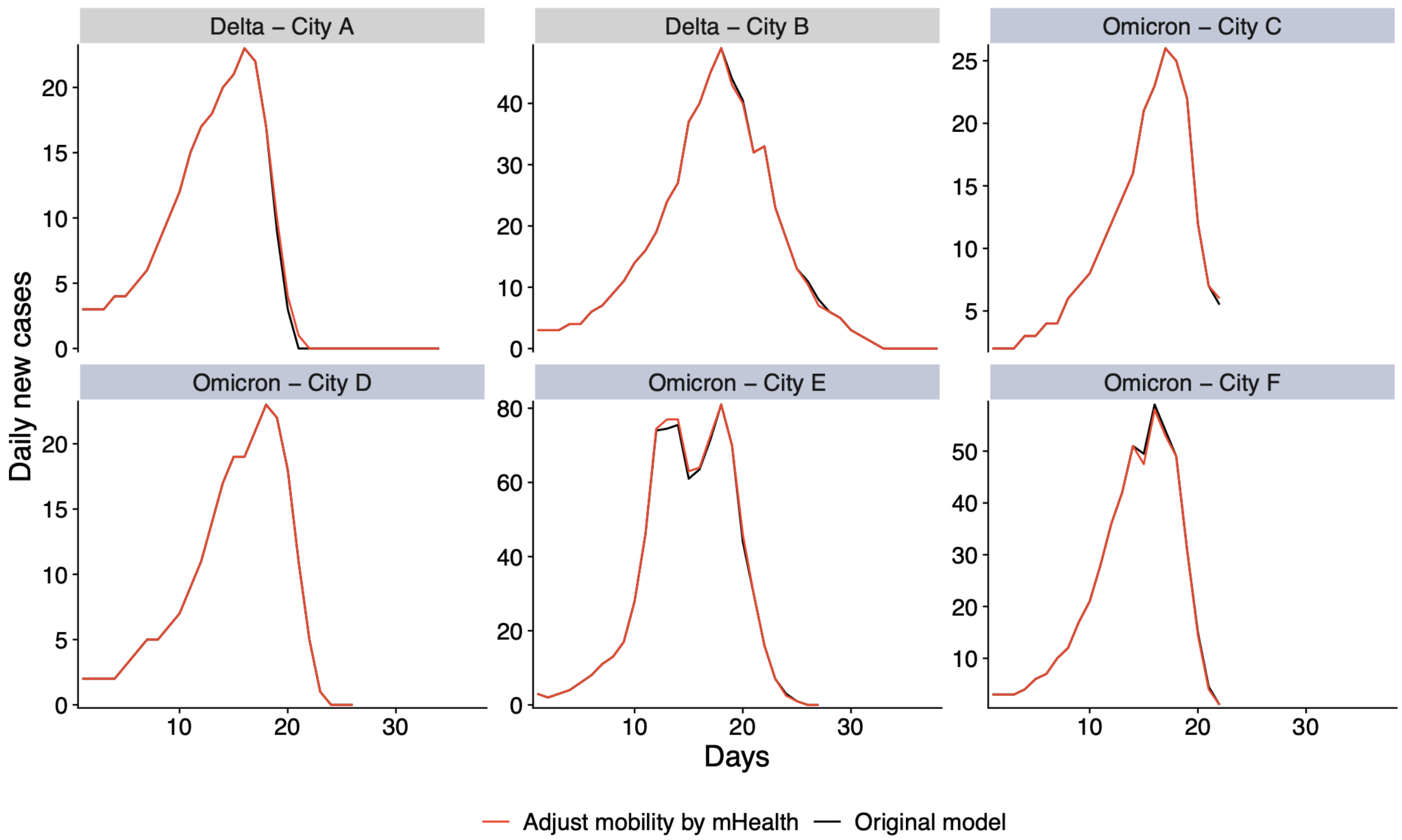

Figure S3. Comparison of model performance between the original specification and an alternative model in which population mobility was adjusted to account for mHealth code-based restrictions.

Table S4. Comparison of estimated parameters between models with and without mobility adjustment based on mHealth code assignment.

**City**	**α (for mobility)**	**λ_{Code}**	**λ_{Code} (Mobility adjusted)**
Delta - City A	0.98 [0.92, 1.00]	0.0005 [0.0000, 0.0010]	0.0004 [0.0000, 0.0010]
Delta - City B	0.98 [0.92, 1.00]	0.0009 [0.0000, 0.0014]	0.0008 [0.0000, 0.0014]
Omicron - City C	0.33 [0.28, 0.37]	0.0009 [0.0000, 0.0024]	0.0008 [0.0000, 0.0027]
Omicron - City D	0.38 [0.33, 0.44]	0.0170 [0.0090, 0.0235]	0.0160 [0.0090, 0.0233]
Omicron - City E	0.83 [0.72, 0.96]	0.0063 [0.0012, 0.0208]	0.0074 [0.0018, 0.0193]
Omicron - City F	0.41 [0.36, 0.48]	0.0034 [0.0013, 0.0066]	0.0034 [0.0015, 0.0065]

3.3 Influence of human mobility on estimated mHealth effects

We examined whether the temporal incorporation of mobility restrictions affected the estimated effects of mHealth interventions. To do so, we applied a within-outbreak interrupted time series modelling approach with lag structures, systematically delaying the mobility data by 1, 4, and 7 days, respectively. The results show that postponing mobility data would induce worse daily infection estimates compared with the original model (Supplementary Fig. S4).

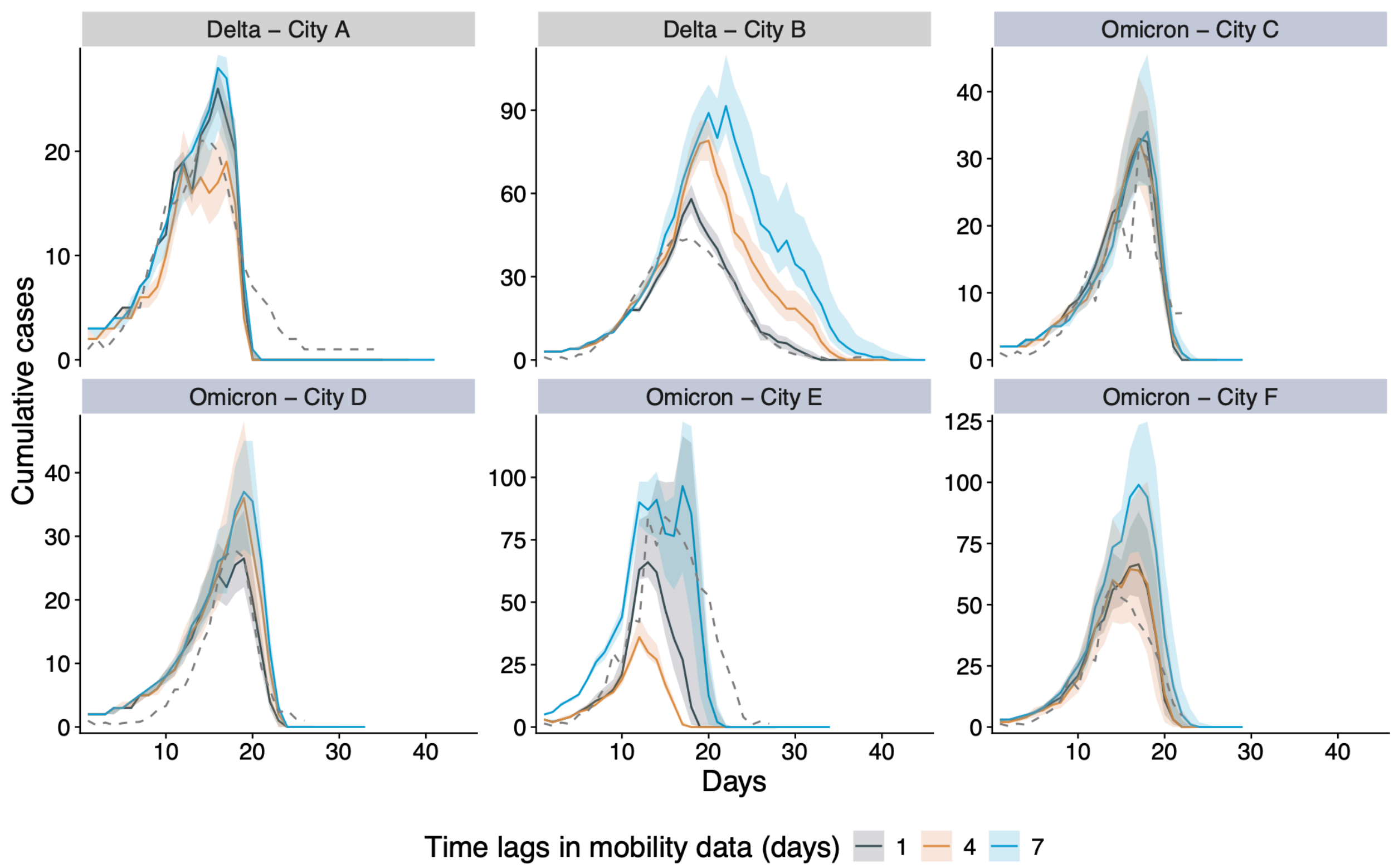

Figure S4. Estimated daily new infections under model specifications incorporating population mobility data with time lags of one, four, and seven days.

We further refitted the model with lagged mobility data. Although most scenarios could still achieve comparable goodness-of-fit as main results, there were substantial changes in the estimated parameters of mHealth code λ_{Code} (Supplementary Fig. S5 and Table S5), indicating

that the potential temporal conflation/collinearity between human mobility and mHealth code effects might be minimal. These results further support the robustness of the two-step modelling framework in distinguishing the contributions of population-wide mobility reductions and targeted digital interventions.

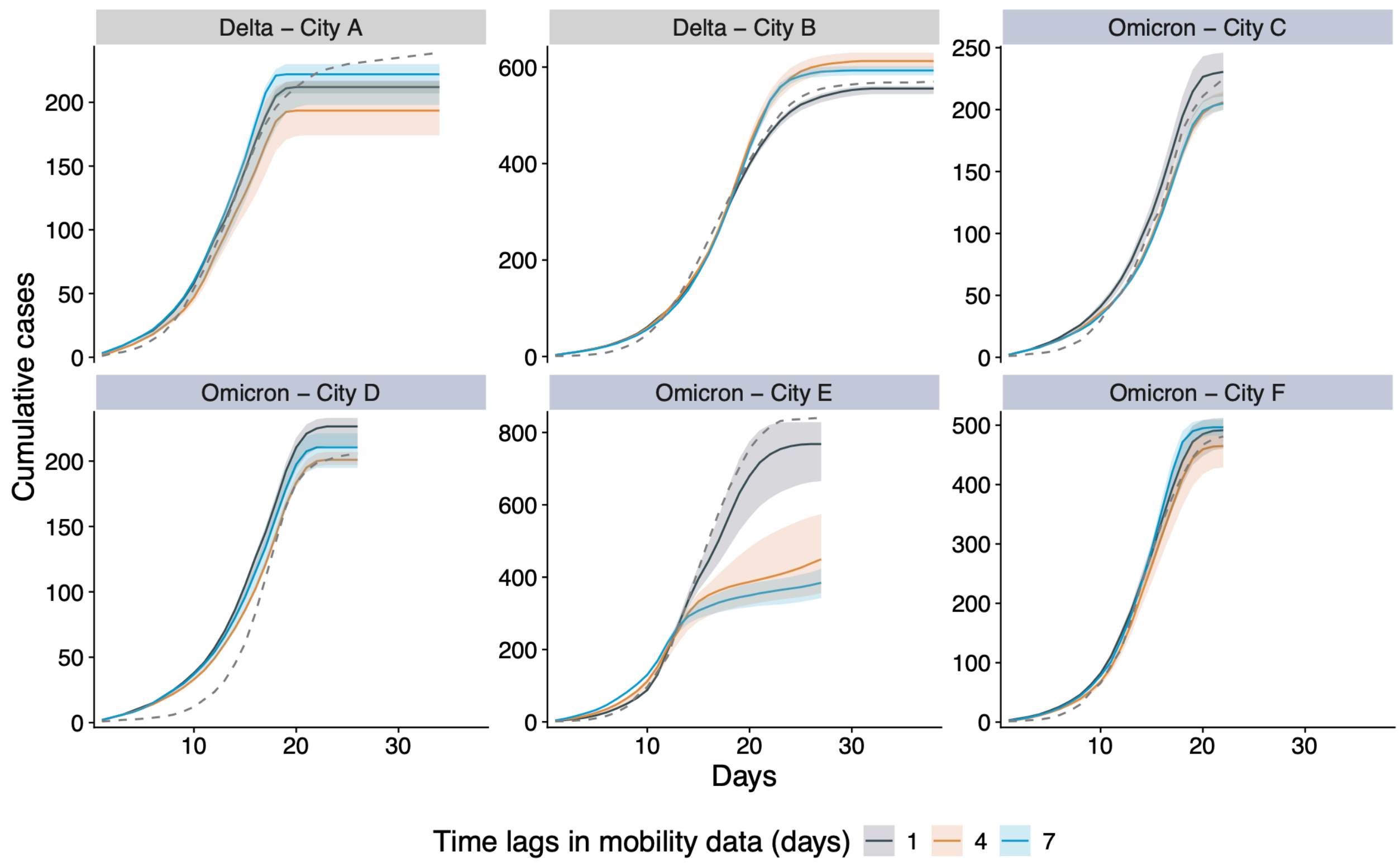

Figure S5. Estimated cumulative infections by postponing population mobility data with time lags of one, four, and seven days and then refitting the model.

Table S5. Comparison of estimated parameters between models fitted using population mobility data with time lags of zero, one, four, and seven days.

Outbreak	Delay in Mobility (days)	α (for mobility)	λ_{Code}
Delta – City A	0	0.983 [0.925, 1.000]	0.0005 [0.0000, 0.0010]
	1	0.950 [0.875, 1.000]	0.0006 [0.0000, 0.0009]
	4	0.985 [0.920, 1.000]	0.0009 [0.0001, 0.0020]
	7	0.980 [0.920, 1.000]	0.0010 [0.0002, 0.0020]
Delta – City B	0	0.982 [0.919, 1.000]	0.0009 [0.0000, 0.0014]
	1	0.955 [0.875, 1.000]	0.0009 [0.0005, 0.0011]
	4	0.926 [0.840, 1.000]	0.0025 [0.0009, 0.0039]
	7	0.904 [0.830, 0.975]	0.0043 [0.0030, 0.0062]
Omicron – City C	0	0.331 [0.285, 0.370]	0.0009 [0.0000, 0.0024]
	1	0.323 [0.290, 0.370]	0.0050 [0.0020, 0.0093]
	4	0.315 [0.280, 0.355]	0.0042 [0.0018, 0.0078]
	7	0.321 [0.285, 0.350]	0.0055 [0.0030, 0.0088]
Omicron – City D	0	0.375 [0.330, 0.440]	0.0170 [0.0090, 0.0235]
	1	0.370 [0.330, 0.420]	0.0175 [0.0092, 0.0300]
	4	0.350 [0.310, 0.395]	0.0157 [0.0084, 0.0200]
	7	0.356 [0.320, 0.395]	0.0254 [0.0146, 0.0406]
Omicron – City E	0	0.832 [0.720, 0.960]	0.0063 [0.0012, 0.0208]
	1	0.856 [0.730, 0.970]	0.0035 [0.0000, 0.0094]
	4	0.888 [0.780, 1.000]	0.0007 [0.0000, 0.0045]
	7	0.756 [0.665, 0.870]	0.0001 [0.0000, 0.0002]
Omicron – City F	0	0.415 [0.360, 0.480]	0.0034 [0.0013, 0.0066]
	1	0.403 [0.365, 0.450]	0.0035 [0.0011, 0.0083]
	4	0.403 [0.350, 0.460]	0.0030 [0.0008, 0.0080]
	7	0.408 [0.345, 0.475]	0.0072 [0.0015, 0.0162]

Supplementary Note 4. Comparison of spillover infections with and without mHealth implementation

The projected spillover infections in the absence of mHealth codes were derived by removing mHealth codes from all cities (Fig. 5). The corresponding daily new infections under this scenario can be found in Supplementary Fig. S6.

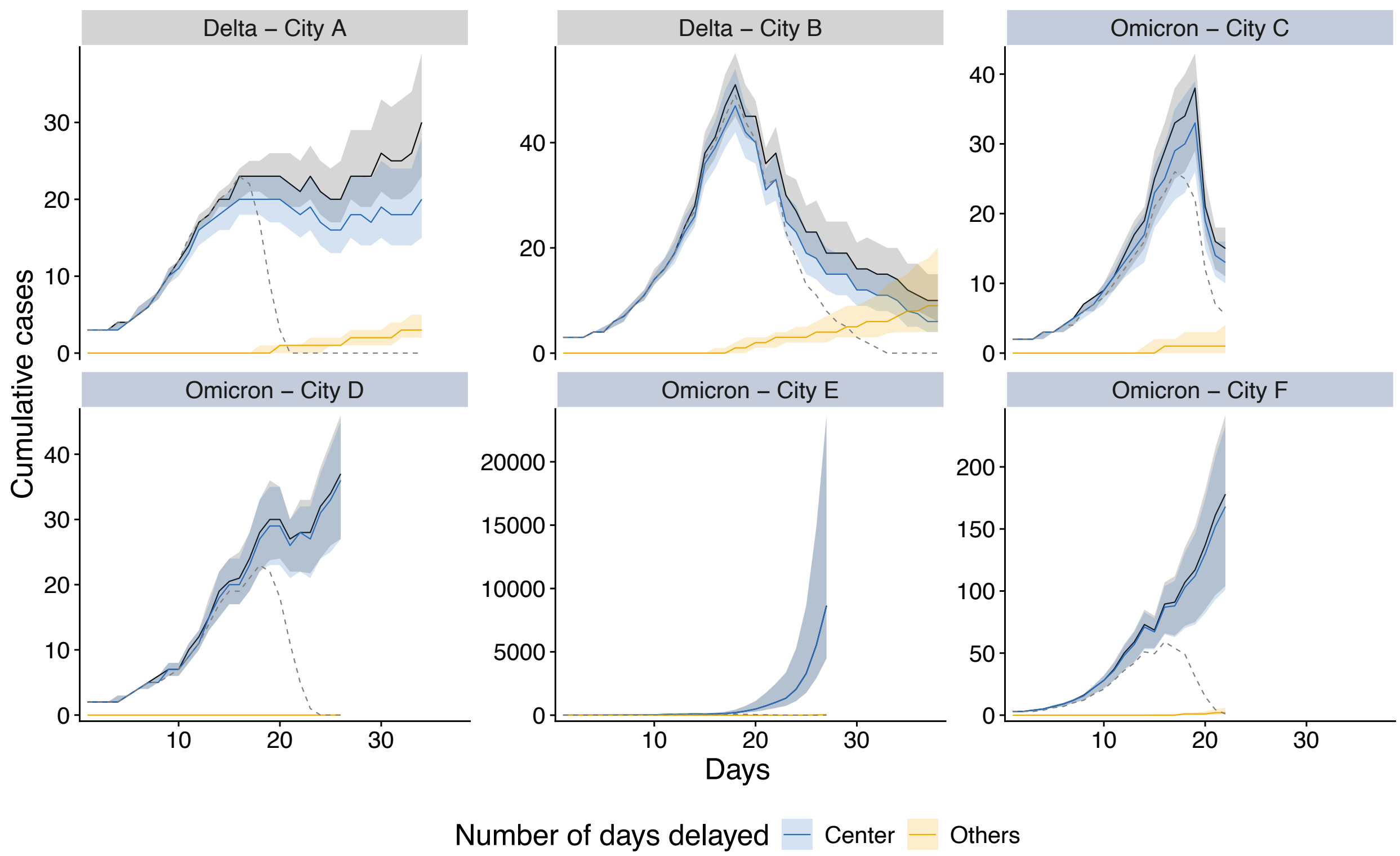

Figure S6. Daily new infections under the counterfactual scenario of geographic spillover in the absence of mHealth codes.

To estimate the net effect of mHealth codes, we compared this counterfactual scenario with actual transmission patterns. We refitted the geographic spillover pattern of each outbreak by integrating traditional contact tracing records and mHealth data of all cities. The estimated curves show few spillover infections across most outbreaks (Fig. S7) and fewer surrounding cities see local epidemics (Fig. S8), which matches the reality. This demonstrated that the

projected infections in Fig. 5 could be largely attributed to the absence of mHealth implementation.

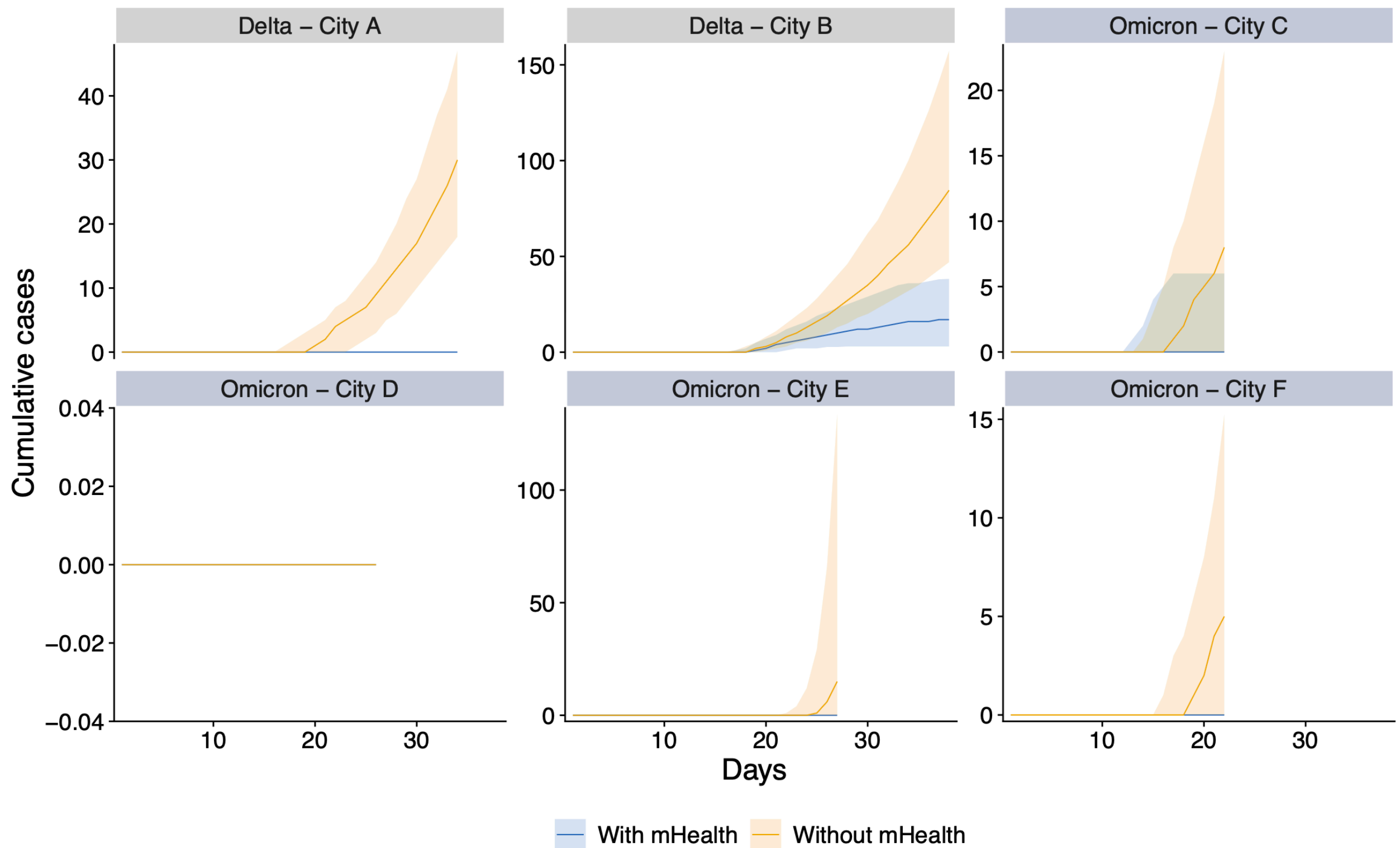

Figure S7. Comparison between spillover infections in surrounding cities with and without mHealth code data included.

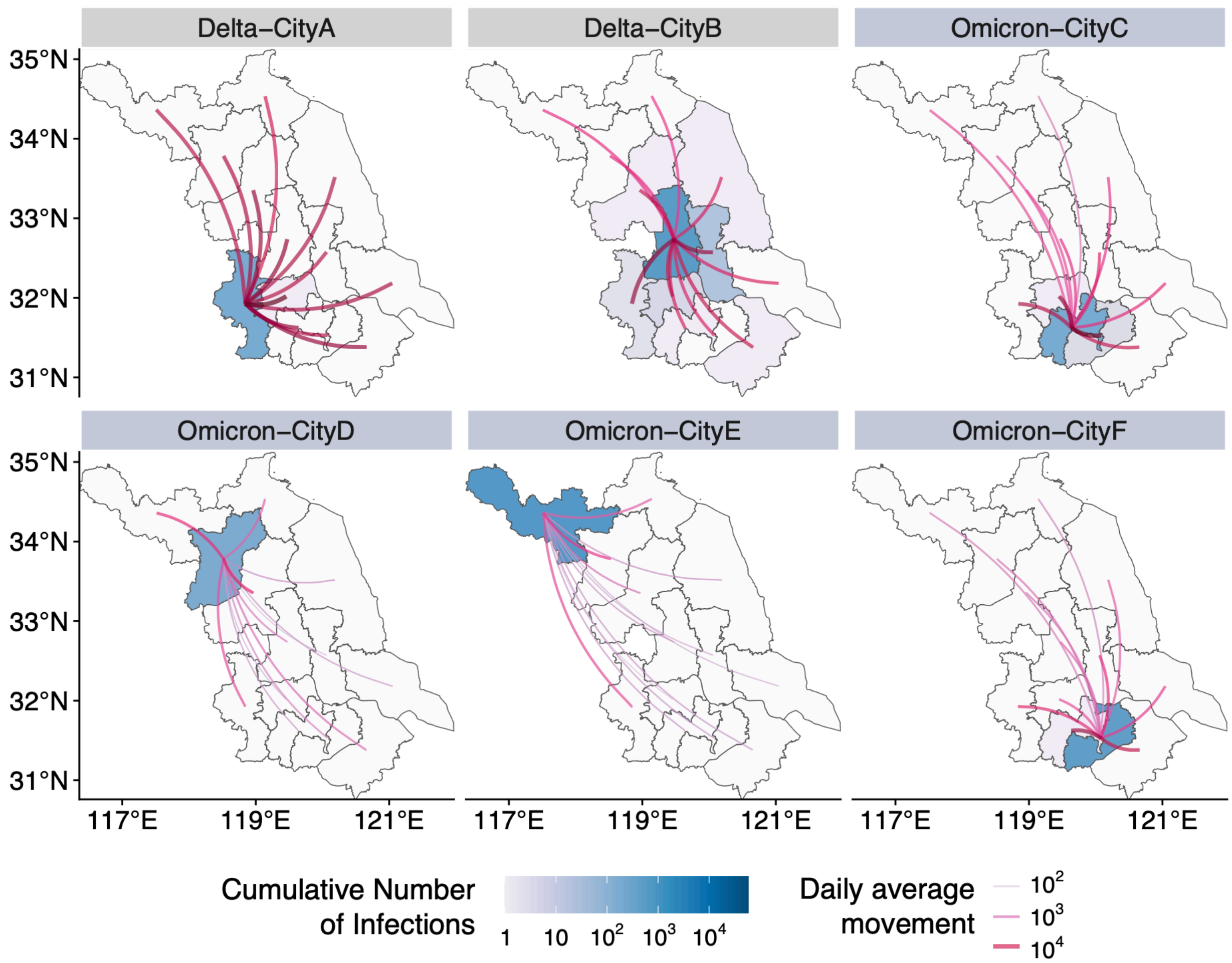

Figure S8. Actual scenario simulations of geographic spillover with mHealth codes data from all cities included.

Supplementary Note 5. Delayed mHealth implementation and robustness test of simulated continued contact tracing

We performed scenario simulations for delayed mHealth code by postponing the mHealth implementation for one day to a week, with modelling time extended accordingly (Fig. S9). Compared with the no-mHealth scenario, the epidemics are more likely to be controlled, though cumulative infections would grow nonlinearly with longer delays. Specifically, for both Delta and Omicron, epidemics are bearable to a maximum of three-day delay in mHealth implementation - curves for different cities are likely to level off during the period of the actual outbreak and the cumulative number of infected cases will not exceed twice the actual number of cases at most, except for City E seeing five times increase with larger uncertainty (5.20 [IQR: 0.38, 91.58]). However, A delay of five days or more cannot always guarantee zero out new infections. We see new infections continue to grow towards the end of actual outbreaks in City E and F when facing more infectious Omicron strains.

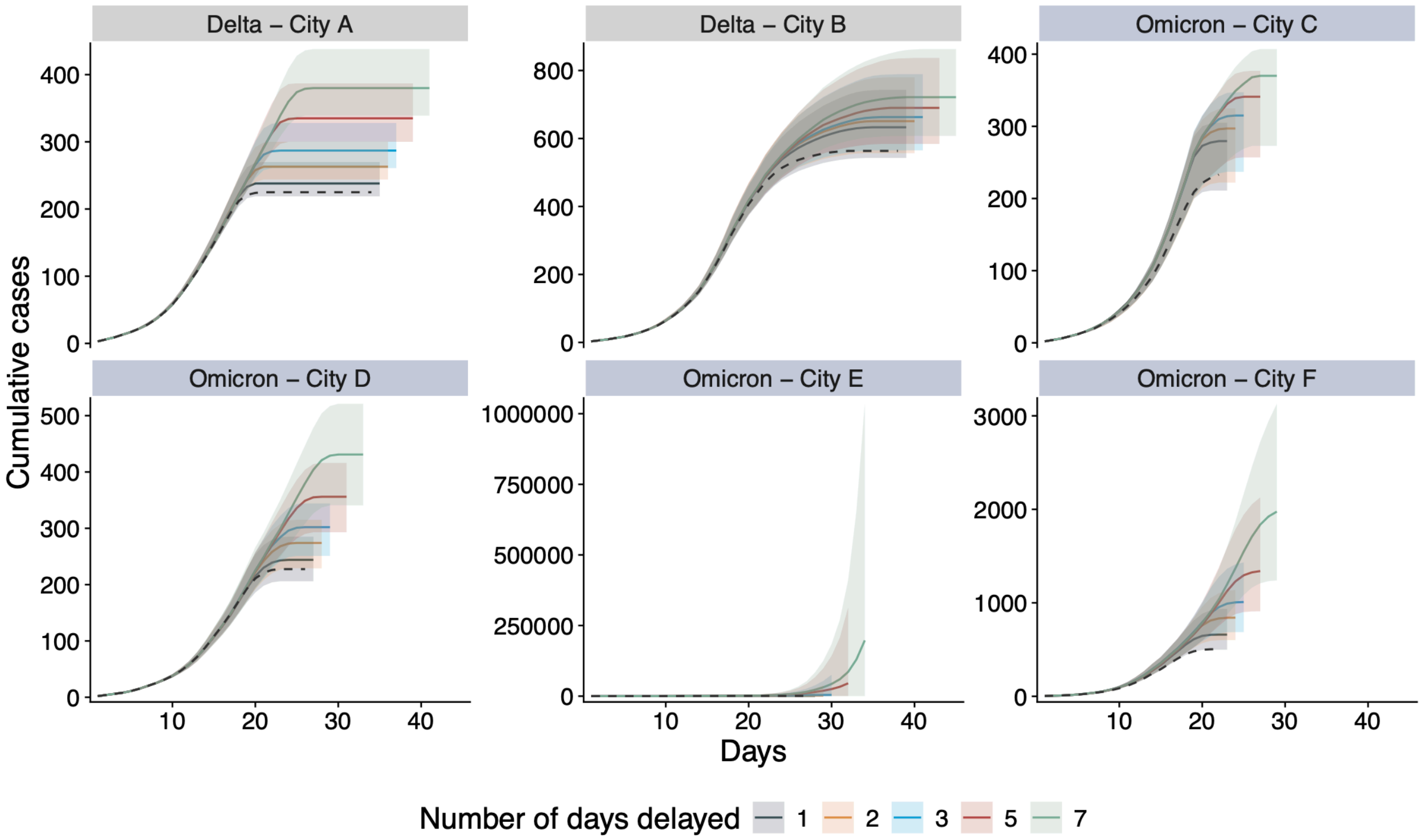

Figure S9. Scenario simulation for delayed mHealth implementation without continued contact tracing.

To avoid overestimating mHealth effects, we considered a more realistic scenario where traditional epidemiological investigation was assumed to continue during the outbreak. We replicated the number of close contacts to the lagging date of the delayed mHealth implementation. To further reflect human resource constraints, we assumed this number would gradually decrease by 25% per day as outbreaks progressed, with an upper limit of 15 contacts identified per reported case on any given day (the provincial CDC indicated this average limit). The estimates in Fig. S10 show similar patterns to the case without continued contact tracing, except for City B having estimates lower than reality. This exception could be attributed to the fact that City B has already seen citywide mobility reduction (lower $\beta'_{0,t}$ comparable to Omicron outbreaks, Fig. 4b) and has conducted intensive epidemiological investigation with a relatively

higher number of close contacts per confirmed cases across Delta outbreaks (7.4 close contacts per case on average). Even one-day replication of close contacts could easily approach the theoretical limit, potentially overestimating the role of traditional investigation.

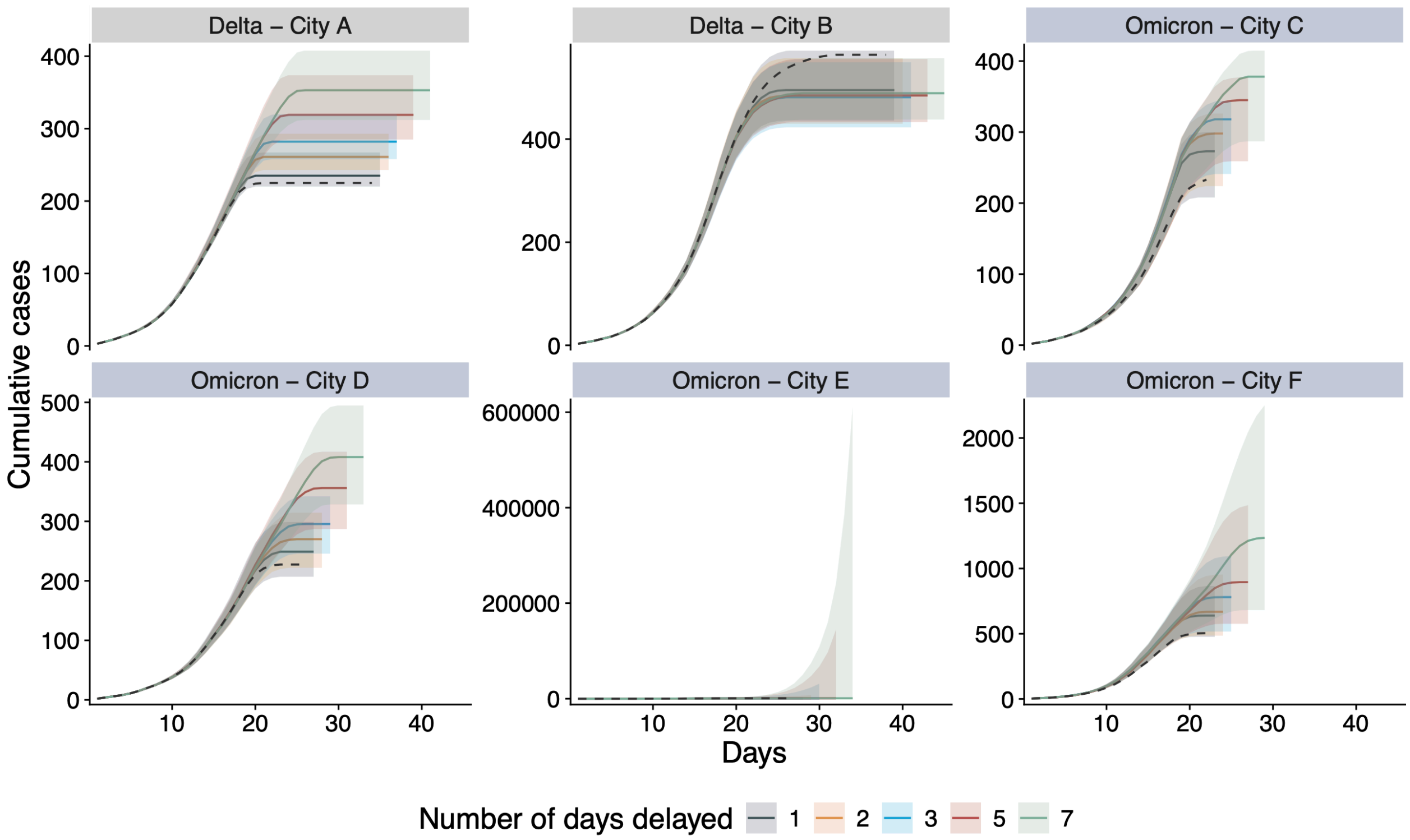

Figure S10. Scenario simulation for delayed mHealth implementation with continued contact tracing.
